# COX-2 expression in mammary invasive micropapillary carcinoma is associated with prognostic factors and acts as a potential therapeutic target in comparative oncology

**DOI:** 10.3389/fvets.2022.983110

**Published:** 2022-09-12

**Authors:** Thaynan Cunha Vieira, Evelyn Ane Oliveira, Bárbara Jaime dos Santos, Fernanda Rezende Souza, Emerson Soares Veloso, Cristiana Buzelin Nunes, Helen Lima Del Puerto, Geovanni Dantas Cassali

**Affiliations:** ^1^Laboratory of Comparative Oncology, Institute of Biological Sciences, Department of General Pathology, Universidade Federal de Minas Gerais, Belo Horizonte, Brazil; ^2^Laboratory of Breast Pathology, Medical School, Department of Pathological Anatomy, Universidade Federal de Minas Gerais, Belo Horizonte, Brazil; ^3^Laboratory of Cellular Behavior, Institute of Biological Sciences, Department of General Pathology, Universidade Federal de Minas Gerais, Belo Horizonte, Brazil

**Keywords:** Breast, comparative oncology, COX-2, immunohistochemistry, mRNA, human, canine model

## Abstract

Pure human and canine mammary invasive micropapillary carcinoma is a rare malignant epithelial tumor accounting for 0.9 to 2% of all invasive mammary carcinomas and present a high rate of lymphatic invasion and metastasis, with unfavorable prognosis. Surgery and chemotherapy are standard treatments for almost all mammary cancer in both species, as well as hormonal and target therapies available for human patients. However, depending on the patient's clinical staging, satisfactory therapeutic results for invasive micropapillary carcinoma are a challenge due to its high capacity of invasion and metastasis. Cyclooxygenase-2 (COX-2) isoform is an important enzyme stimulated by cytokines, growth factors and oncogenes activation to synthetizes prostaglandins in inflammatory process. COX-2 overexpression is associated with angiogenesis and invasion and contributes to cancer development, disease progression, tumor recurrence and regional lymph node metastasis in human and canine mammary carcinomas. This enzyme can be targeted by non-steroidal anti-inflammatory drugs and its inhibition can reduce tumor growth and metastasis in several cancer types. Given the similarity between both species, the present study aims to elucidate the involvement of COX-2 mRNA and protein expression in canine (cIMPC) and human (hIMPC) pure invasive mammary micropapillary carcinoma, with clinicopathological and survival data. Twenty-nine cases of cIMPC and 17 cases of hIMPC were analyzed regarding histologic type, grade, age, tumor size, lymph node condition, extracapsular extension, inflammatory infiltrate and immunophenotype. When available, information on adjuvant treatment, recurrence, metastasis and overall survival were collected. The present study demonstrated COX-2 protein expression in 65.5% of cIMPC and 92.3% of hIMPC, and an association with more advanced histological grades in bitches and higher Ki67 in women. COX-2 mRNA expression was significantly higher in cIMPC than in hIMPC, and its expression was not associated with COX-2 protein expression in both species. COX-2 mRNA expression was associated with negative-ER hIMPC as well as higher Ki67. cIMPC demonstrated proportional early development, more regional metastasis, and a prevalence of negative estrogen receptor, than hIMPC. This is the first time COX-2 expression is associated with negative prognostic factors in both cIMPC and hIMPC, besides the overexpression of COX-2 protein in such unfavorable histological type, which suggests that COX-2 can act as a potential target in IMPC.

## Introduction

Women represent the largest piece of the population in the development of breast cancer, with an estimated 66,280 new cases in Brazil for the triennium 2020-2022 and 287,850 new cases in the United States in 2022 (1–3). In addition to this high incidence, breast cancer is not only considered the most prevalent type of cancer in women, with the exception of non-melanoma skin cancer, but also caused about 14.23 deaths per 100,000 women in Brazil in 2019 (4). Likewise, mammary neoplasms in female dogs represent about 50% of tumors in the species, being almost 80% considered malignant (5–10). Malignant neoplasms of the mammary gland are one of the best studied cancers in comparative oncology due to the similarities between dogs and humans with regard to spontaneous tumor incidence, age of development, genetic factors, hormonal influence, biological behavior and environmental factors involved in both species (11–13).

Pure human breast invasive micropapillary carcinoma (hIMPC) is a rare malignant epithelial tumor which accounts for 0.9 to 2% of all human invasive breast carcinomas and shows a high rate of lymphatic invasion and metastasis, with unfavorable prognosis due to recurrences and axillary lymph node metastasis at the time of diagnosis (14–16). This histologic type was first described in a dog by Cassali and colleagues in 2002 (17) and followed by other description (18). Posteriorly, canine invasive micropapillary carcinoma (cIMPC) was recognized and incorporated in standard classifications for canine mammary tumors (19, 20). Studies describe the low incidence (about 2% of all canine malignant mammary tumors) and aggressive biological behavior similarities in both species, highlighting regional and distant metastasis at the time of diagnosis and low survival rates in canine patients with IMPC (11, 12, 17, 19–23).

Although the pure form of IMPC is considered rare in both species, the combination with other histological types are also observed, with micropapillary areas found in about 7.4% of all invasive human breast cancers (14, 21).

In both species, surgery and chemotherapy are the most common standard treatments for breast cancer, as well as hormonal and target therapies available for human patients. However, depending on the patient's clinical staging, satisfactory therapeutic results for invasive micropapillary carcinoma are a challenge due to its high capacity of invasion and metastasis (24–26).

Cyclooxygenases (COX) enzymes are essential in physiological processes due to the conversion of arachidonic acid in prostaglandins. Such enzymes are constituent of health tissues (COX-1) or induced by pathological events such as inflammation (COX-2) (27, 28). Overexpression of COX-2 is described in several canine and human tumors, including breast cancer, and is associated with disease progression and poor prognostic parameters such as histologic type, tumor recurrence and regional lymph node metastasis (28–31). In this context, COX-2 isoform is stimulated by cytokines, growth factors and oncogenes activation and plays a key role in cancer development through proliferation, mutagenesis, angiogenesis, immunological modulation and invasion properties (32–36). On the other side, COX-2 can be targeted by non-steroidal anti-inflammatory drugs (NSAIDs) and its inhibition, alone or in combination with other therapies, can reduce tumor growth and metastasis in experimental and canine comparative models for human breast cancer, as well as *in vitro* studies (32, 37–44). Selective COX-2 inhibition was described in gastric, colorectal, lung, skin cancers, esophageal squamous cell carcinoma, nasopharyngeal carcinoma and malignant glioma with antiangiogenic and antimetastatic capacities (41–46).

COX-2 expression in canine and human mammary tumor subtypes with aggressive behavior, such as IMPC for both species, is still not fully elucidated and needs to be investigated. Therefore, to clarify the expression of this enzyme and its potential as a new therapeutic target in rare and aggressive tumors, the present study aims to evaluate the association of COX-2 mRNA and protein expressions with clinicopathologic parameters and overall survival in cIMPC and hIMPC.

## Materials and methods

### Canine sample collection

Twenty-nine cases of pure cIMPC were selected from 2011 to 2021 at the Laboratory of Comparative Pathology at UFMG, Brazil. The formalin-fixed paraffin-embedded (FFPE) samples were obtained from simple, regional and unilateral or bilateral radical mastectomies excisional biopsies. Data on the patient's age, breed, tumor location, tumor size, lymph node status, extracapsular extension and associated tumors in adjacent mammary glands, when present, were collected through histopathological records. Tumor size, obtained from formalin-fixed samples not considering skin or subcutaneous tissue, was considered the largest measure when the three dimensions of the nodule were available (height x length x width) and classified according to TNM clinical staging criteria: T1 (<3 cm), T2 (3–5 cm), and T3 (>5 cm) (47, 48). Information about reproductive status, treatment, recurrence and metastasis were accessed through telephone contact with the responsible veterinarian of each case. Five FFPE samples of normal adjacent mammary glands were obtained from biopsies of patients submitted to a radical mastectomy and diagnosed only with carcinoma in mixed tumors (...) in other adjacent mammary glands (...) with no lymph node metastasis as control.

### Human sample collection

Seventeen cases of pure hIMPC were selected from 2010 to 2020 at the Laboratory of Breast Pathology at Medical School of UFMG, Brazil. The FFPE samples were obtained from biopsies of mastectomies, setorectomies, and tumorectomies, with lymph nodes evaluation when available. No samples were from patients who received radiation therapy, hormonal therapy, or chemotherapy before the surgery. Data of patient's age, tumor location, tumor size, lymph node status and extracapsular extension, when present, were collected through histopathological records. Tumor size was considered the largest measure when the three dimensions of the nodule were available (height x length x width) and classified according to TNM clinical staging criteria: T1 (<2 cm), T2 (2–5 cm) and T3 (>5 cm) (24, 49). Four FFPE samples of normal mammary glands were obtained from biopsies of esthetical surgeries and used as control.

### Histopathology

The histological slides stained with Hematoxylin and Eosin (H&E) were from fragments of neoplasms fixed in 10% neutral buffered formalin and embedded in paraffin. The cIMPC were classified based on Consensus for the diagnosis, prognosis and treatment of canine mammary tumors (20) and according to Goldschimdt et al. (19), while hIMPC were classified as described by WHO Human Histological Classification Criteria (14). All cases were reviewed by experienced pathologists specialized in canine mammary gland tumors (G.D.C.) and human breast tumors (C.B.N.). Only pure cIMPC (>75% of micropapillary pattern) and hIMPC (>90% of micropapillary pattern) were included in the present study (14, 21). According to the Nottingham system, the histological grade for both species was determined through morphologic features such as tubule formation, nuclear pleomorphism, and mitotic count, a value from 1 (favorable) to 3 (unfavorable) was assigned for each feature, and the total scores for all three categories were used to establish grades I (3–5), II (6–7) and III (8–9), as previously described (20, 24, 50–52).

### Tumor inflammatory infiltrate

The analysis was carried out in 4 μm H&E stained histological sections and the inflammatory infiltrate was classified by cell type and the distribution was carried out in peripheric and intratumor areas, as suggested by Estrela-Lima (53) and Mohammed (54). The cIMPC inflammatory distribution was evaluated in peripheral and intratumor areas and classified as: focal, when present 1 to 3 inflammatory foci were present; multifocal, when more than 3 inflammatory foci were present; and diffuse, when inflammatory cells were evenly distributed in the tumor section. Similarly, inflammatory distribution in hIMPC was defined also in both areas and classified as: score 0, when absence of inflammatory cells; score 1, when mild and patchy inflammatory cells were present; score 2, when prominent inflammatory reactions were observed; and score 3, when dense inflammatory infiltrates with destruction/compression of cancer cells was present. In the present study, to facilitate comparative analysis, scores 1, 2, and 3 were represented by focal, multifocal and diffuse nomenclature, respectively.

### Immunohistochemistry

Canine sample sections of 4 μm-thick were mounted on gelatin-coated slides and submitted to the streptavidin-biotin-peroxidase complex method with commercial detection anti-mouse/anti-rabbit system (Novolink Polymer Detection SystemTM; Leica Biosystems, Newcastle, United Kingdom) according to the manufacturer's instructions. Reagents were applied manually, and the antibody reactions were visualized by incubating the slides for 3 min with chromogen 3,3'-diaminobenzidine tetrachloride (DAB) diluent (Dako North America, *Via* Carpinteria, United States). The samples were washed in distilled water for 5 min, then counterstained with hematoxylin. Details of the antibodies, dilutions, antigen retrieval procedures, and incubation times used are given in Table 1.

**Table 1:** Details of immunohistochemical reagents and methods used in the study for cIMPC samples.

**Target antigen**	**Clone**	**Manufacturer**	**Dilution**	**AR method**	**Incubation**
ER	EP1	Dako	Ready to use	Pressurized heating (125°C)	16h/4°C
PR	NCL-L-PGR	Novocastra	1:50	Pressurized heating (125°C)	16h/4°C
HER2	Polyclonal	Dako	1:200	Pressurized heating (125°C)	16h/4°C
Ki67	MIB-1	Dako	1:50	Pressurized heating (125°C)	16h/4°C
COX-2	SP21	Thermo System	Ready to use	Pressurized heating (125°C)	16h/4°C

AR, antigen retrieval; ER, estrogen receptor; PR, progesterone receptor.

Human sample sections of 4 μm-thick were mounted on gelatin-coated slides and submitted to the streptavidin-biotin-peroxidase complex method with commercial detection anti-mouse/anti-rabbit system (EnVisionTM; Dako North America, *Via* Carpinteria, United States) according to the manufacturer's instructions. Reagents were applied manually, and the antibody reactions were visualized by incubating the slides for 5 min with chromogen 3,3'- diaminobenzidine tetrachloride (DAB) diluent (Dako North America, *Via* Carpinteria, United States). The samples were washed in distilled water for 5 min, then counterstained with hematoxylin. Details of the antibodies, dilutions, antigen retrieval procedures, and incubation times used are given in Table 2.

**Table 2:** Details of immunohistochemical reagents and methods used in the study for hIMPC samples.

**Target antigen**	**Clone**	**Manufacturer**	**Dilution**	**AR method**	**Incubation**
ER	1D5	Dako	1:200	Pressurized heating (125°C)	1h/room temperature
PR	SP2	Dako	1:200	Pressurized heating (125°C)	1h/room temperature
HER2	Polyclonal	Dako	1:180	Pressurized heating (125°C)	1h/room temperature
Ki67	MIB-1	Dako	1:180	Pressurized heating (125°C)	1h/room temperature
COX-2	SP21	Thermo System	Ready to use	Pressurized heating (125°C)	1h/room temperature

AR, antigen retrieval; ER, estrogen receptor; PR, progesterone receptor.

As previously described in the literature, interpretation criteria were established for canine and human samples. Immunoreactivity for canine and human ER and PR was considered positive when more than 1% of the nuclei of neoplastic cells expressed those markers (24, 55, 56). For the interpretation of HER2 staining, the standard established by the American Society of Clinical Oncology/American College of Pathologists was used for canine and human samples, in which complete and robust membrane staining in more than 10% of cells is considered positive (57–59). The proliferative index was calculated by counting the number of nuclei positive for Ki-67 staining in a total of 500 neoplastic cells from each lesion and classified as low (<15%), intermediate (16 to 30%) and high (>30%) immunostaining (60–65). The final semi-quantitative analysis of COX-2 was conducted for both species and estimated by multiplying a distribution of staining value with a value for intensity of staining, which ranges from 0 to 12 (30, 66). Values for staining intensity were given from 0 to 3, being that 0 means no labeling (-), 1 is equivalent to weak staining (+), 2 is moderate staining (+ +), and 3 is strong intensity (+ + +). The number of positive cells was analyzed in five fields (40x) for all tumors, in which a distribution value between 0 and 4 was obtained, where 0 means 0%, 1 represents < 10% of labeled cells, 2 stands for 10 to 30%, 3 to 31 to 60%, and 4 indicates more than 61% of labeled cells. COX-2 positive protein expression in cIMPC was considered when samples showed at least a weak immunostaining, while negative protein expression was considered when no immunostaining was observed (66). In a second analysis, in a comparative analyze, COX-2 scores from 0 to 5 were considered low and scores from 6 to 12 were considered high for both species, as proposed by Lavalle et al. (66). For hIMPC cases, samples were considered positive for COX-2 when a moderate to strong intensity was identified, and negative when an absent or weak immunostaining intensity was observed, as previously suggested in the literature (30).

### Immunophenotype

Establishment of immunophenotype for cIMPC followed that proposed by Nunes (67), which classifies tumors as Luminal A (ER or PR +, HER2 – and Ki67 <20%), Luminal B (ER or PR +, HER2 – and Ki67 >20%), Luminal B HER2+ (ER or PR +, HER2 + and any Ki67), HER2 (ER -, PR -, HER2 +, any Ki67) or Triple-Negative (ER -, PR -, HER2 - and any Ki67). Similarly, the immunophenotype for hIMPC followed that proposed in St. Gallen International Breast Cancer Conference Expert Panel (68, 69), which considers Luminal A (ER +, PR + ≥20%, HER2 - and Ki67 <14%), Luminal B (ER +, PR <20% or Ki67 >14%, and HER2 -), Luminal B HER2+ (ER +, any PR, HER2 + and any Ki67), HER2 (ER -, PR -, HER2 + and any Ki67), and Triple-Negative (ER -, PR -, HER2 – and any Ki67).

### RNA extraction and real-time quantitative PCR

Formalin-fixed paraffin-embedded (FFPE) tissue samples of cIMPC, hIMPC, and normal mammary samples of each species were cut into 80 μm sections in sterile and endonuclease-free microtubes, and endonucleases were removed from surfaces and during microtome sections with RNase Away (Ambion^®^). RNA was isolated from FFPE tissue samples with RecoverAll^TM^ Total Nucleic Acid Isolation Kit (Thermo Fisher Scientific, Walthan, MA, United States, Code AM1975), with minor modifications to the manufacturer's instructions. The deparaffinization was done twice, the protease digestion was performed under incubation of 1h at 50 °C and 10 min at 80 °C, and the elution was done with 60 μl of DEPC-Treated Water (Thermo Fisher Scientific, Walthan, MA, United States). Extracted RNAs were quantified, and 260/280 nm absorbance was determined by NanoDrop Nucleic Acid Quantification (Thermo Fisher Scientific, Walthan, MA, United Sates).

Total RNA was reverse transcribed with High-Capacity cDNA Reverse Transcription Kit and RNase Inhibitor (Thermo Fisher Scientific, Wilmington, Delaware, United States) according to the manufacturer's instructions, using 140 ng and 100 ng of total RNA for canine and human samples, respectively. For qPCR, 2 μl of cDNA and 1.5 mM of each forward and reverse primer were used in a final 30 μl qPCR reaction with Power SYBRGreen PCR Master Mix Kit (Invitrogen, CA, United States) and duplicate samples were carried out in a 7,500 SDS Real-Time PCR machine (Thermo Fisher Scientific, Wilmington, Delaware, United States). Real-time PCR thermal cycling conditions were as follows: (1) one cycle of 50°C/2 min; (2) one cycle at 95°C/10 min; (3) 45 cycles of 95°C/15s, followed by a melting curve of 58°C. Primer sequences used in qPCR amplification for canine and human COX-2, canine reference gene HPRT and human reference gene GAPDH are listed in Table 3.

**Table 3:** Primers sequences for qPCR.

**Target**	**Specie**	**Nucleotide sequences**	**Fragment size**	**GenBank**
HPRT For	Canis	5'- CCTTGGTCAAGGAGCATAATC−3'	140	NM_001003354.1
HPRT Rev		5'- GTCAAGGGCATATCCTACAAC−3'		
COX-2 For	Canis	5'- TCAAGGGAGTCTGGAACA - 3'	86	NM_001003354.1
COX-2 Rev		5' - CAAATGTGACCGGGATGT - 3'		
GAPDH For	Sapiens	5' -TGGGTGTGAACCATGAGAAG- 3'	125	NM_001289746.1
GAPDH Rev		5' -GAGTCCTTCCACGATACCAAAG- 3'		
COX-2 For	Sapiens	5'-TACCCGGACAGGATTCTATG-3'	93	NM_000963.3
COX-2 Rev		5'-TGCACTGTGTTTGGAGTG-3'		

COX-2 gene expression data were accessed by comparative CT method, as the reference gene was used to normalize COX-2 gene expression and generate dCt [(CT target gene)—(CT reference gene)]. The 2^−dCt^ was calculated and applied to obtain COX-2 relative expression by comparing the IMPC group with the control group (normal mammary glands), as proposed by Schmittgen and Livak (70).

### Survival analysis

Information on canine survival was obtained *via* telephone contact with the responsible veterinarian of each case. The overall survival was calculated from the date of surgery to the date of patient death. Deaths unrelated to the tumor were censored. Information on human estimated survival was obtained through the data system available. Overall survival was calculated from the date of surgery to the last date in which the patient was active in the follow-ups. Patients that were alive until the later date of follow-up were censored.

### Statistical analysis

GraphPad Prism version 8.0 (GraphPad Software, La Jolla, CA, United States) was used to conduct statistical analyses and the significance level was set to *p* ≤ 0.05. The Shapiro-Wilk test evaluated data distribution. For quantitative results, means were compared with Mann-Whitney or unpaired *t*-test, depending on the normality of data distribution. Possible correlations were assessed using Spearman's or Person's tests. Relationships between qualitative variables were investigated with Chi-square or Fisher's exact test. Survival curves were estimated through the Kaplan-Meier method and differences in survival between groups were compared with the log-rank test.

## Results

### Clinicopathological characteristics

Clinicopathological characteristics of canine and human cases are detailed above and shown in Table 4.

**Table 4:** Details of the clinicopathological characteristics of cIMPC and hIMPC.

	**cIMPC**	**hIMPC**
Mean age (years)	11.3 ± 2.8	52.4 ± 12.4
**Tumor size**		
T1	37% (10/27)	31.3% (5/16)
T2	22.2% (6/27)	62.5% (10/16)
T3	40.7% (11/27)	6.3% (1/16)
**Tumor growth**		
Plaque	38.5% (10/26)	0
Nodular	61.5% (16/26)	100% (16/16)
**Lymph node metastasis**		
Absent	4.8% (1/21)	36.4% (4/11)
Present	95.2% (20/21)	63.6% (7/11)
**Extracapsular extension**		
Absente	65% (13/20)	71.4% (5/7)
Present	35% (7/20)	28.6% (2/7)
**Histologic grade**		
I	11.1% (3/27)	0
II	70.4% (19/27)	43.8% (7/16)
III	18.5% (5/27)	56.3% (9/16)
**Inflammatory infiltrate**		
**Peripheric area**		
Focal	72.4% (21/29)	12.5% (2/16)
Multifocal	27.6% (8/29)	87.5% (14/16)
Difuse	0	0
**Intratumor area**	
Focal	3.5% (1/29)	47.1% (8/17)
Multifocal	79.3% (23/29)	52.9% (9/17)
Difuse	17.2% (5/29)	0
**ER protein expression**		
Negative	86.2% (25/29)	18.7% (3/16)
Positive	13.8% (4/29)	81.3% (13/16)
**PR protein expression**		
Negative	0	37.5% (6/16)
Positive	100% (29/29)	62.5% (10/16)
**HER2 protein expression**		
Negative	86.2% (25/29)	53.3% (8/15)
Positive	13.8% (4/29)	46.7% (7/15)
**Ki67 protein level**		
Low	0	46.1% (3/13)
Intermediate	3.5% (1/29)	69.2% (9/13)
High	96.5% (28/29)	7.7% (1/13)
**COX-2 protein expression**		
Negative	34.5% (10/29)	7,7% (1/13)
Positive	65.5% (19/29)	92.3% (12/13)
**Immunophenotype**		
Luminal A	0	15.4% (2/13)
Luminal B	86.2% (25/29)	30.7% (4/13)
Luminal B HER2+	13.8% (4/29)	38.5% (5/13)
HER2	0	15.4% (2/13)
Triple-Negative	0	0
**MST (months)**		
Total	4.1	58.5

cIMPC, canine invasive micropapillary carcinoma; hIMPC, human invasive micropapillary carcinoma.

Canine and human patients were represented by female and women in all cases, respectively. The ages of canine patients at the time of surgery ranged from 6 to 16 years (11.3 ± 2.8), while the ages of human patients ranged from 35 to 77 years (52.4 ± 12.4). Mixed Breed Dogs (34.6%; 9/26) and Poodle (30.8%; 8/26) were the most affected breeds. Canine tumor location was more frequently identified in inguinal mammary glands, which represented 34.5% (10/29) of cases. The most frequent tumor size in cIMPC was T3 with 40.7% (11/27) of cases, followed by T1 with 37% (10/27) and T2 with 22.2% (6/27), and tumor growth with plaque pattern was present in 38.5% (10/26) of cases. In hIMPC, the most frequent tumor size was T2, which accounted for 62.5% (10/16) of cases, followed by T1 with 31.3% (5/16) and T3 with 6.3% (1/16).

For 11 canine patients (37.9%), follow-ups were available. Previously neutered animals accounted for 54.5% (6/11) of cases, while intact animals until tumor excision represented 45.5% (5/11). Surgery alone was instituted as treatment in 36.4% (4/11) of cases, surgery and chemotherapy (doxorubicin, carboplatin or cyclophosphamide) also in 36.4% (4/11), surgery and complementary therapy (*viscum album* or non-steroidal anti-inflammatory drugs) in 18.1% (2/11), and no information in 9.1% (1/11) of cases. No recurrence was identified. However, systemic metastasis after tumor excision occurred in 54.5% (6/11) of cases. Metastasis was diagnosed in bones of pelvic members, abdominal cavity, distant lymph nodes, and lungs. Pelvic limb edema was a clinical sign identified in 27.3% (3/11) of cases, associated with lymph node enlargement and impaired lymphatic drainage.

Metastasis to regional lymph nodes, accessed with tumor excision, occurred in 95.2% (20/21) of cIMPC cases and in 63.6% (7/11) of hIMPC cases. Only 1 canine patient did not present metastasis to regional lymph nodes at the time of diagnosis. Among cIMPC and hIMPC cases with regional metastasis, 35% (7/20) and 28.6% (2/7) presented extracapsular extension, respectively. Associated tumors diagnosed in patients with cIMPC, but in other mammary glands, included others cIMPC in 27.6% (8/29) of cases, carcinoma in mixed tumors in 24.1% (7/29), solid carcinomas in 17.2% (5/29), papillary carcinomas in 13.8% (4/29), benign mixed tumors in 6.9% (2/29), and basaloid carcinoma, tubular carcinoma, pleomorphic lobular carcinoma and malignant adenomyoepithelioma in 3.4% each (1/29, each).

### Histopathology

**Figure 1:**
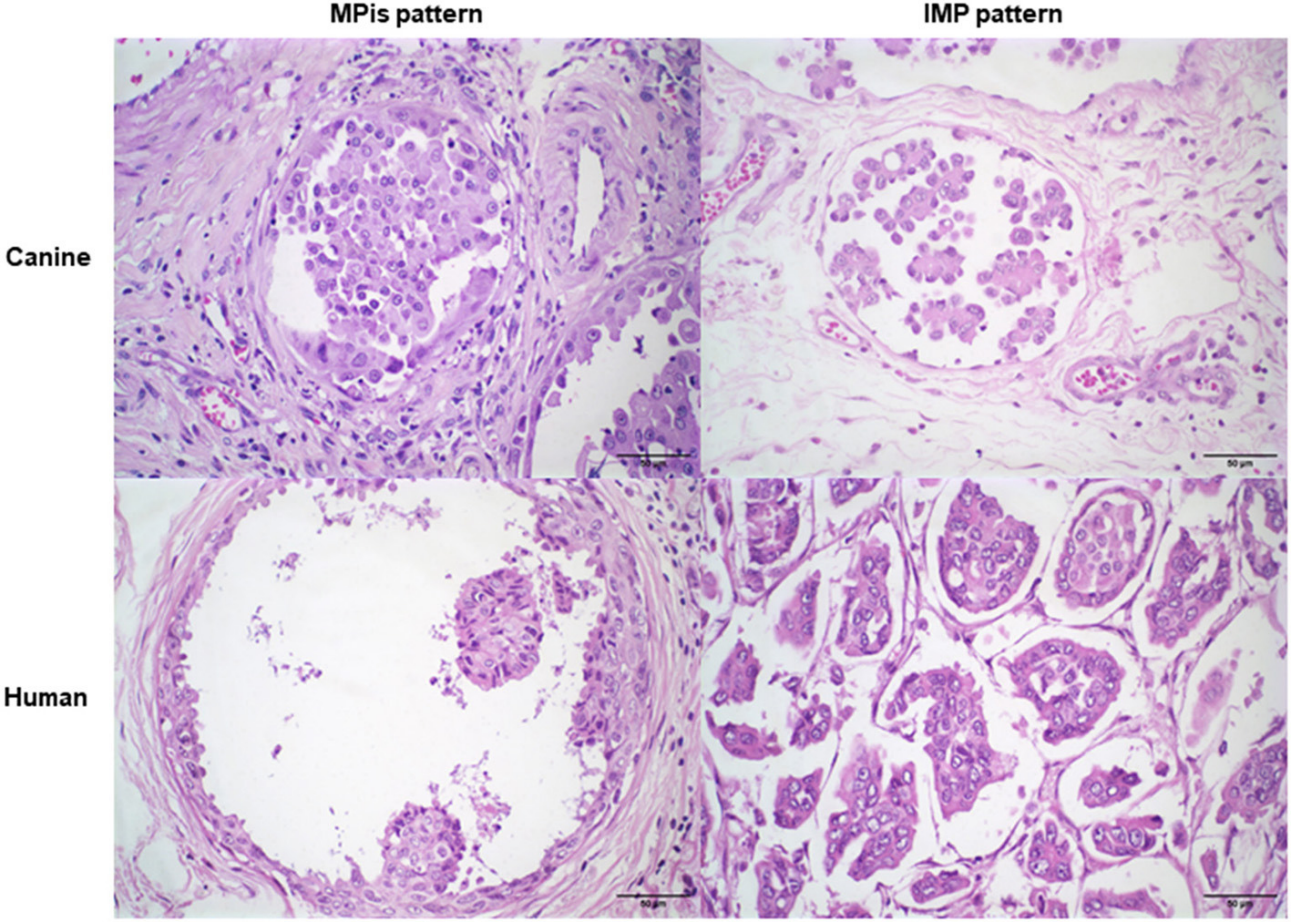
Morphological characteristics of cIMPC and hIMPC. Micropapillary in situ (MPis) pattern with empty spaces surrounded by tumor epithelial cells. Invasive micropapillary (IMP) pattern with tumor cells arraged in “morule-like clusters”, called micropapills with no fibrovascular cores, and surrounded by empty spaces not lined by endothelial, myoepithelial or epithelial cells.

Canine and human samples shared histopathological characteristics of tumor cells arranged in “morule-like clusters,” called micropapills, and surrounded by clear or empty spaces not lined by myoepithelial, epithelial, or endothelial cells, with no fibrovascular cores. Tumor cells exhibited large eosinophilic cytoplasm and variable anisokaryosis and mitotic count. The *in situ* micropapillary areas were evidenced by the empty spaces surrounded by tumor epithelial cells and invasive micropapillary areas were identified by clusters of tumor epithelial cells arranged in empty spaces (Figure 1).

The histologic grade for cIMPC and hIMPC was classified as grade I in 11.1% (3/27) of canine cases, grade II in 70.4% (19/27) and 43.8% (7/16), and grade III in 18.5% (5/27) and 56.3% (9/16) of cases, respectively. Cases that had fewer than 10 invasive micropapillary areas were not histologic graduated.

### Tumor inflammatory infiltrate

The inflammatory infiltrate in cIMPC and hIMPC was more frequently identified with predominance of mononuclear cells, such as lymphocytes, plasma cells and macrophages, and occasionally neutrophils and eosinophils. The cIMPC presented focal distribution in peripheric tumor in 72.4% (21/29) of cases and intratumoral multifocal distribution in 79.3% (23 / 29). In hIMPC, the infiltrate distribution was commonly classified as multifocal in peripheric in 87.5% (14/16) of cases and intratumor in 52.9% (9/17). One case was not classified in the peripheric area due to an incisional biopsy origin.

### RE, RP, HER2, Ki67 and COX-2 protein expressions

**Figure 2:**
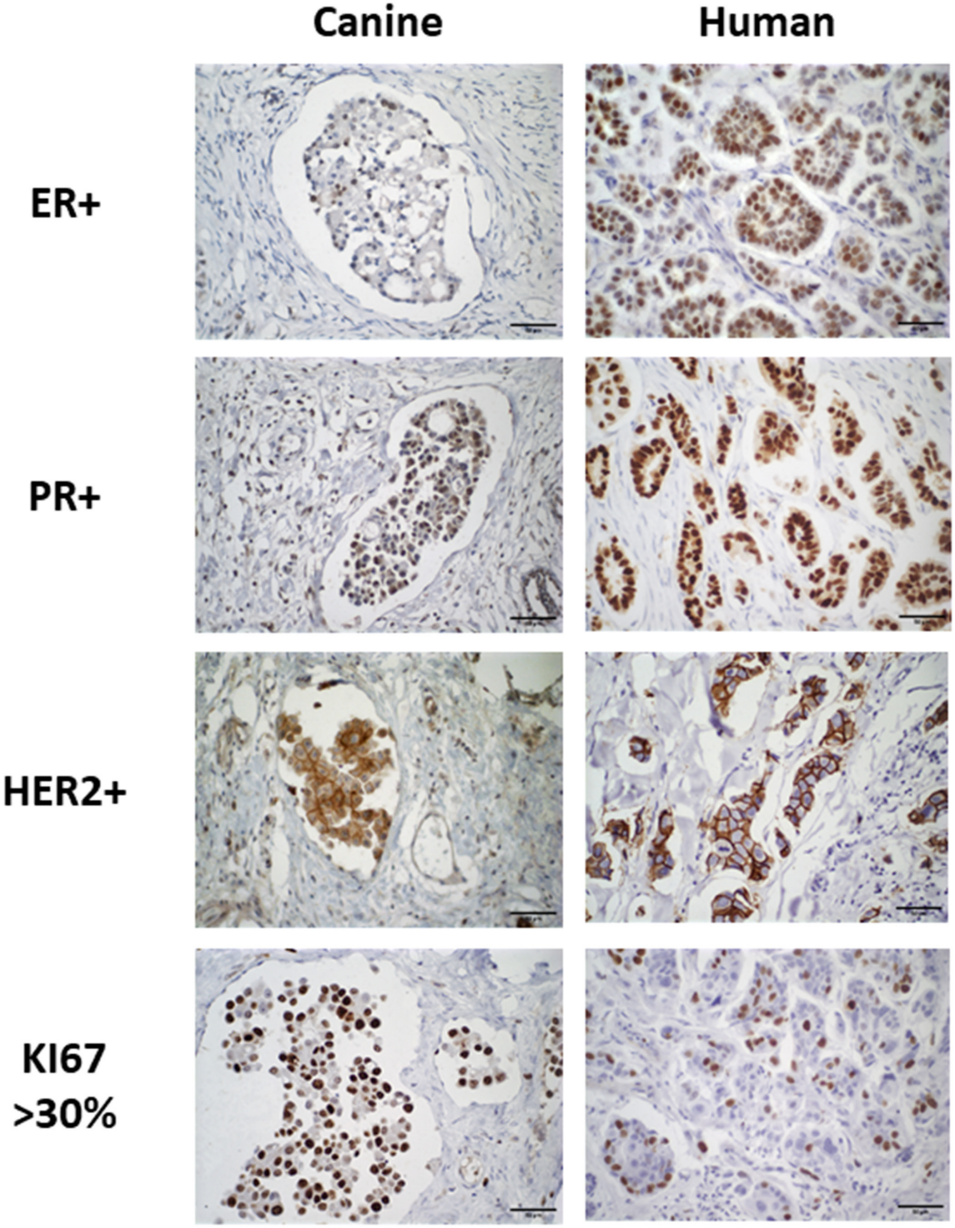
Immunostaining of positive ER, PR, HER2 and high Ki67 levels in cIMPC and hIMPC. ER-positive nuclear immunoexpression in >1% of neoplastic cells in cIMPC and hIMPC. PR-positive nuclear immunoexpression in >1% of neoplastic cells in cIMPC and hIMPC. HER2-positive complete and strong membrane immunoexpression in >10% of neoplastic cells in cIMPC and hIMPC. High Ki67 nuclear immunoexpression in >30% of neoplastic cells in cIMPC and hIMPC.

The cIMPC and hIMPC cases showed ER nuclear positivity in 13.8% (4/29) and 81.3% (13/16) of cases, respectively, while PR nuclear positivity were identified in 100% (29/29) and 62.5% (10/16) of cases, respectively. The oncoprotein HER2 was overexpressed in 13.8% (4/29) of cIMPC and 46.7% (7/15) of hIMPC samples. The proliferation index determined by nuclear staining for Ki67 ranged from 23.22 to 94.49% (64.98 ± 0.19) for cIMPC and 6.95 to 34.69% (21.51 ± 0.08) for hIMPC. The immunostaining for these biomarkers is shown in Figure 2.

**Figure 3:**
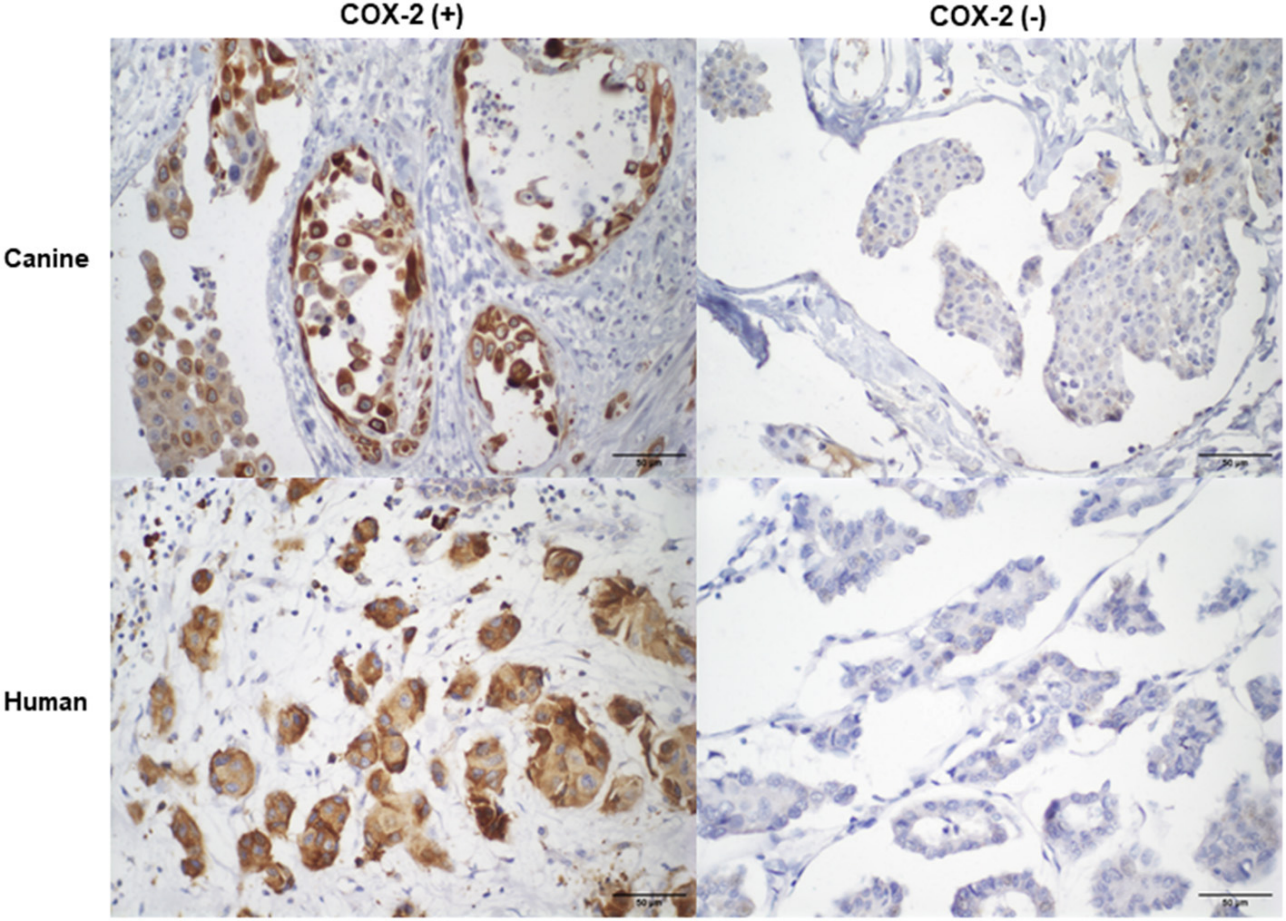
COX-2 immunostaining in cIMPC and hIMPC. Canine and human with positive COX-2 expression with strong cytoplasmatic immunostaining in 10 to 30% of neoplastic cells, totalizing score 6. Canine and human with negative cytoplasmatic COX-2 immunostaining, totalizing score 0.

The COX-2 protein expression was positive in 65.5% (19/29) of cIMPC cases and 92.3% (12/13) of hIMPC. Cytoplasmatic immunostaining intensity for COX-2 was absent in 34.5% (10/29) and 7.7% (1/13) of cIMPC and hIMPC cases, respectively (Figure 3). No association was observed between low COX-2 (scores 0 to 5) and high COX-2 (scores 6–12) with all parameters analyzed in cIMPC and hIMPC.

Weak (+) and moderate (++) intensity were observed only in cIMPC samples, in 6.9% (2/29) and 10.3% (3/29) of cases, respectively. Strong (+ + +) intensity was predominant in cIMPC and hIMPC, in 48.3% (14/29) and 92.3% (12/13) of samples, respectively. The distribution of cytoplasmatic immunostaining in cIMPC was more observed with value 2 (10 to 30%) in 41.4% (12/29) of cases, followed by value 4 (more than 61%) in 10.3% (3 / 29), and values 1 (<10%) and 3 (31 to 60%), in 6.9% each (2/29, each). For hIMPC, the distribution was also more evident with value 2 in 46.2% (6/13) of cases, followed by value 1 in 38.5% (5/13), and value 3 in 7.7% (1/13).

COX-2 score, obtained by multiplying the intensity and distribution, ranged from 0 to 12 in cIMPC and from 0 to 9 in hIMPC. The cIMPC showed prevalence of score 6 in 37.9% (11/29) of cases, followed by scores 0 in 34.5% (10/29), 4 and 9 in 6.9% each (2/29, each), and 1, 2, 8, and 12 in 3.4% each (1/29, each). The hIMPC also demonstrated prevalence of score 6 in 20.7% (6/13) of cases, followed by scores 3 in 38.5% (5/13), and 0 and 9 in 7.7% each (1/13, each). According to the level of COX-2 positivy, 48.3% (14/29) of cIMPC and 46.2% (6/13) of hIMPC were considered with low COX-2, and 51.7% (15/29) of cIMPC and 53.8% (7/13) of hIMPC, was high COX-2.

### Immunophenotype

Canine samples were classified as Luminal B in 86.2% (25/29) of cases, followed by Luminal B HER2+ in 13.8% (4/29). For hIMPC, Luminal B HER2+ was identified in 38.5% (5/13) of cases, followed by Luminal B in 30.7% (4/13), Luminal A and HER2 in 15.4% of cases, each (2/13, each).

### COX-2 mRNA expression

**Figure 4:**
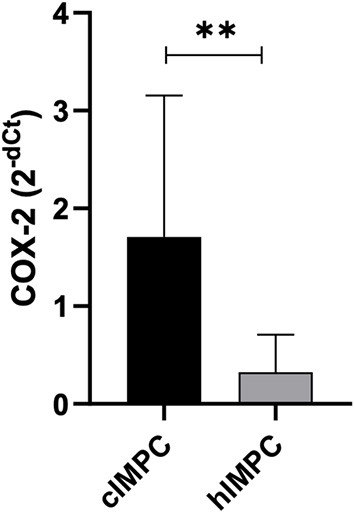
COX-2 mRNA expression in cIMPC and hIMPC. Relative Quantification (RQ) of COX-2 mRNA expression in cIMPC (*n* = 9) and hIMPC (*n* = 10) using HPRT and GAPDH as the reference gene. COX-2 gene expression data were accessed by comparative Ct method, using the 2^−*dCt*^ method to obtain COX-2 relative expression by comparing the IMPC group with the control group (normal mammary glands). Bars represent mean fold expression with SEM. The difference between groups was determined by comparing delta Ct. Mean values of the IMPC vs. healthy controls (**p < 0.005).

The median COX-2 mRNA expression showed no significant difference when IMPC and control groups were compared in canine (*p* = 0.08) and human (*p* > 0.9999) samples. The cIMPC showed higher COX-2 mRNA expression compared to hIMPC (*p* = 0.0031) (Figure 4). Association between median COX-2 mRNA expression for negative and positive COX-2 protein expression in cIMPC and hIMPC demonstrated no significant difference (*p* = 0.3429 for cIMPC; *p* = 0.2844 for hIMPC).

### COX-2 expression in association with prognostic factors

COX-2 protein expression was positively correlated with higher histologic grades in cIMPC (*p* = 0.0441; *r* = 0.3979). Association between COX-2 mRNA expression and histologic grades in hIMPC was not significant (*p* = 0.2261).

No significative difference was found between COX-2 expression and tumor size either in cIMPC or hIMPC. The median COX-2 mRNA expression compared with tumor size was not significant (*p* = 0.3312)in cIMPC. Similar occurred for hIMPC, in which the difference was not significant (*p* = 0.1778). In addition, comparison of COX-2 mRNA expression and the characteristic of plaque growth in cIMPC showed no significance (*p* = 0.0971).

**Figure 5:**
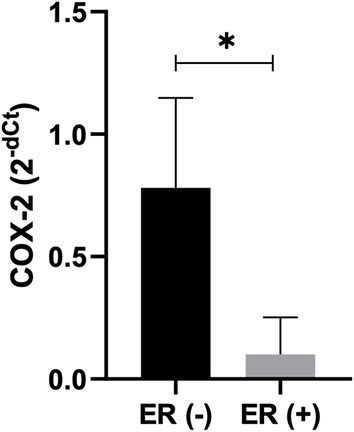
COX-2 mRNA expression correlation with ER expression in hIMPC. Relative Quantification (RQ) of COX-2 mRNA expression in hIMPC (*n* = 10) samples using GAPDH as the reference gene. COX-2 mRNA expression in hIMPC showed significant correlation with negative ER expressions (*p* = 0.0238; *r* = −0.8216) (*p < 0.05).

Higher median COX-2 mRNA expression in hIMPC showed a strong negative correlation with negative ER protein expressions (*p* = 0.0238; *r* = −0.8216) (Figure 5). The same was not observed for cIMPC (*p* > 0.9999), as well as no association of COX-2 protein expression with ER protein expressions in cIMPC (*p* = 0.3694) and hIMPC (*p* = 0.0937).

**Figure 6:**
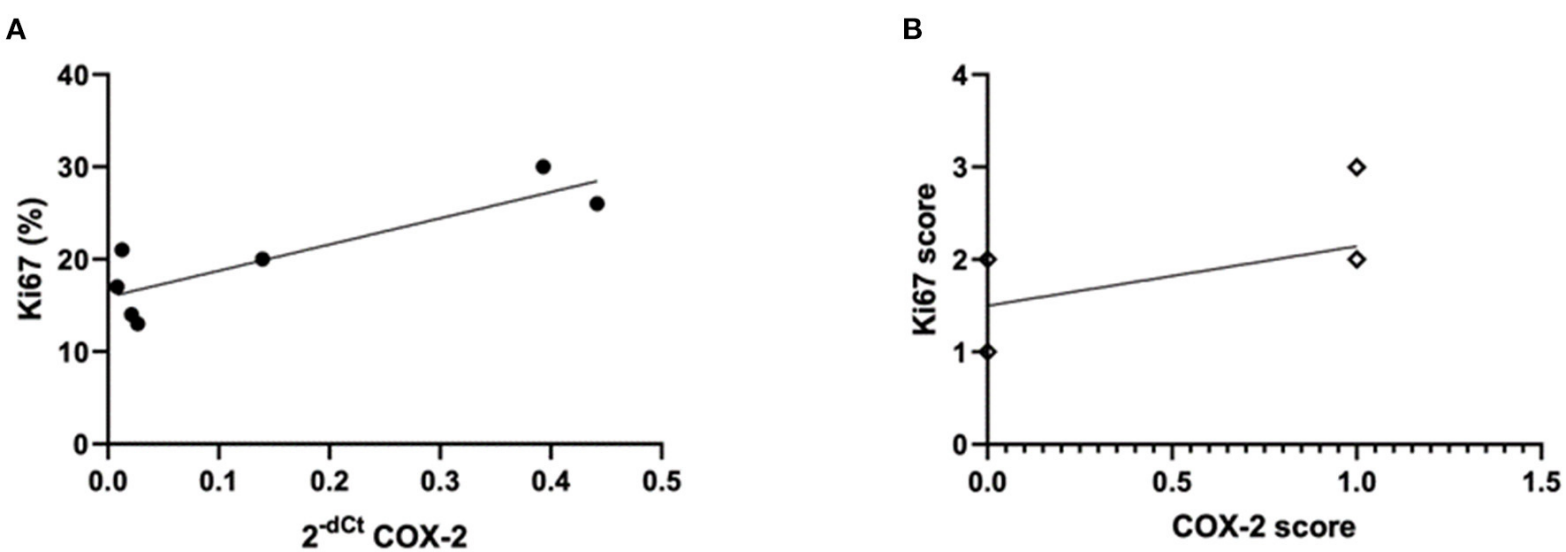
COX-2 mRNA and protein expression and Ki67 expression in hIMPC. **(A)** Relative Quantification (RQ) of COX-2 mRNA expression in hIMPC (*n* = 10) samples using GAPDH as the reference gene. COX-2 mRNA higher expressions were correlated with higher Ki67 levels in hIMPC (*p* = 0.0162; *r* =0.8469) (*p < 0.05). **(B)** Positive COX-2 expression was correlated with higher Ki67 levels in hIMPC (*p* = 0.0490; *r* = 0.6094) (*p < 0.05).

In hIMPC, a correlation was also observed in positive COX-2 protein expressions with higher Ki67 levels (*p* = 0.0490; *r* = 0.6094). Still, higher COX-2 mRNA expressions were correlated with higher Ki67 levels (*p* = 0.0162; *r* = 0.8469) (Figure 6).

### Median survival time

Survival information was available for 44.8% (13/29) of dogs. Of these, 84.6% (11/13) died due to mammary tumor complication, being that euthanasia was performed in 54.5% of those (6/11). Two cases were censored (15.4%) due to deaths related to progressive degenerative articular disease and loss of follow-up contact. The median survival time (MST) for cIMPC was 4.1 months (124.7 days). There was no difference in MST between COX-2 positive and negative protein expressions (*p* = 0.2254), To consider the impact of treated dogs after surgery that could interfere within MST data previous shown, dogs that received adjuvant therapy presented median of 4.7 months (140 days), in contrast to only 0.25 months (7.5 days) in dogs that did not receive adjuvant therapy. However, MST was not significantly different (*p* = 0.5648).

For human patients, estimated survival information was available for 88.2% (15/17) of cases. A total of 26.7% (4/15) of patients had the last follow-up information before the analysis and were considered closed, while 73.3% (11/15) were in currently follow-ups. The MST for hIMPC was 58.5 months (1,753.8 days). Due to the few closed cases, MST was not statistically significant for any variable in hIMPC.

## Discussion

The findings of the present study report, for the first time, the involvement of COX-2 mRNA and protein expressions with other prognostic and predictive factors in cIMPC and hIMPC, as in a comparative approach for this extremely aggressive mammary histologic type. Better detailed above, the increased COX-2 mRNA and protein expressions were associated with higher histologic grades, negative-ER IMPC and higher Ki67 levels, with eventual and important differences between species.

The overall characteristics of canine patients are consistent with previous reports in the literature of dogs with IMPC, such as the average of 11 years, tumor location, high rate of local and distant metastasis and high histological grades (17, 18, 20, 21, 71). Similarly, hIMPC showed concordant clinicopathologic features described in other studies, with median age between 52 and 60 years, high rate of local metastasis and high histological grades (72–74). When canine age is extrapolated to human comparative age, as proposed by Lebeau (75) and Wang (76), cIMPC was developed in equivalent patient's age of 43.8 years, almost one decade before hIMPC patients. Such finding was previously observed in a study that found other clinicopathologic characteristics that suggest the more aggressive biological behavior of IMPC in dogs than in humans (71). The increased COX-2 mRNA expression, which was significantly found in cIMPC than in hIMPC, may contribute to such poor prognosis in the specie and allow further studied to investigate the potential involvement of COX-2 mechanisms in the growth, invasion and metastasis properties in cIMPC.

The ER and PR protein expressions in cIMPC and hIMPC are reported in other studies with IMPC, as a molecular characteristic of this histological type (14, 16, 21, 71, 74, 77, 78). It is worth noting, in the present study, that the ER and PR protein expressions differ from those observed in hIMPC and this result should be interpreted carefully, since IMPC can express or not these hormonal receptors and the limited number of samples can explain this data for cIMPC. The maintenance of hormonal receptors in breast cancer is generally associated with better prognosis. However, IMPC seems to present poor prognosis despite the common presence of ER and PR protein expression (21, 23, 56, 59, 71, 77, 79–81). Such fact suggests this aggressive histologic type may be related to other biological processes involved in its behavior. The overexpression of HER2 protein in IMPC is observed with variable frequencies, reported in 10 to 50% of cases in both species, although prognostic and predictive significance in canine mammary tumors and IMPC are still uncertain (14, 21, 59, 71, 74, 77, 78, 82, 83). Considered as a negative prognostic factor, high Ki67 protein levels is involved in proliferation, invasion and metastasis process in breast cancer (84). In addition, high Ki67 protein levels can predict a better chemotherapy response (60, 85–87). Due to these immunoexpressions in cIMPC and hIMPC, the most frequent immunophenotype for both species were Luminal B and Luminal B HER2+, with less frequency of non-luminal subtypes, in accordance with previous studies (14, 74, 88, 89). The high frequency of multifocal inflammatory infiltrate, predominantly with mononuclear cells, in cIMPC and hIMPC in this study was also described in the literature in association with increased lymph node and distant metastasis, and consequently poorer prognosis (90, 91).

High frequency of COX-2 protein expression was observed in cIMPC and hIMPC cases. Interestingly, the compromise of some statistical analysis because only one hIMPC was negative in COX-2 protein expression, implies such enzyme is highly expressed in this aggressive histologic type. In general, COX-2 protein expression is observed in almost 40% of invasive breast carcinomas in humans, which emphasizes the potential role of COX-2 involvement in IMPC to be further invetigated (31). Despite COX-2 protein expression being considerably studied in canine mammary tumors, no specific information on frequency in cIMPC is available (25, 44, 66). As previous demonstrated in canine mammary tumors, higher COX-2 protein expression was associated with worse MST, on the other side, they are more prone to respond to COX-2 inhibitors (38, 40–42, 44, 92). In this study, no significant difference was observed in the MST of the low and high COX-2 groups in cIMPC and hIMPC. However, the patients were heterogeneous and underwent different therapeutic managements, including NSAIDs, which may interfere with these results due to the median of 7, 5 and 140 days for patients with no treatment and with treatment, respectively. For cIMPC, MST of 4.1 months (124 days) is in concordance with previous studies (21, 71). The hIMPC patients had MST of 58.4 months (1,753 days), only evaluated with 4 cases, since almost all the patients diagnosed after 2014 are still active in follow-ups. Studies show almost 60% of patients have 5 years of MST, with 48% of patients coming to 10 years of survival (74, 93).

The COX-2 mRNA expression tends to be upregulated in cIMPC tumors with grade III, in tumors with larger sizes in cIMPC and hIMPC, as well as in tumors with plaque growth in cIMPC, which highlights the potential correlation with these prognostic markers in the further analysis due to the increase of COX-2 protein expression. These prognostic markers are associated with IMPC, but not yet with possible COX-2 status in IMPC (10, 14, 20, 31, 71, 84, 94). A significant association found with higher COX-2 mRNA expression in negative-ER and in higher Ki67 protein levels in hIMPC, as well as the COX-2 protein expression also related with higher Ki67 protein levels in hIMPC, sustaining the COX-2 involvement in breast tumors with poor characteristics (28, 31, 32). Similarly, the positive association of COX-2 protein expression with higher histologic grades in cIMPC is also in agreement with previous studies in canine mammary tumors (20, 21, 71). The fact there was no significance in COX-2 mRNA expression in comparison with COX-2 positive and negative protein expression in IMPC, of both species, suggests the identification of protein instead of mRNA is more important as a potential therapeutic target, as also shown in previous study that analyzed different histologic types of breast cancers (95). This mechanism may be involved in post-transcriptional physiological factors, such as miRNAs inhibiting COX-2 protein expression due to repressive capacities (96–99). When deregulated in the cancer context, this post-transcriptional factor is altered and no more effective in avoiding protein synthesis (96, 98, 100).

Despite this study allow the association of COX-2 expression with important established prognostic and predictive factors for IMPC, some limitations are need to be highlighted. Although the pure form is considered rare and our study counted with 29 cIMPC and 17 hIMPC cases from retrospective 10 years, the difficult to access and obtain complete follow-ups from all patients limited a more robust prognostic analysis.

In conclusion, this is the first time COX-2 expression is associated with negative prognostic factors in both cIMPC and hIMPC, such as higher histologic grades, negative-ER and high Ki67 levels in IMPC. In addition, this study demonstrates the high frequency of COX-2 protein expression in this unfavorable histological type, which suggests COX-2 as a potential target in IMPC to contribute to better outcomes in canine and human patients.

## Data availability statement

The raw data supporting the conclusions of this article will be made available by the authors, without undue reservation.

## Ethics statement

The studies involving human participants were reviewed and approved by Human Ethics Committee from the Universidade Federal de Minas Gerais (CEUA/UFMG), under protocol number CAAE 43947521.3.0000.5149. Written informed consent for participation was not required for this study in accordance with the national legislation and the institutional requirements. The animal study was reviewed and approved by Ethics Committee on the Use of Animals from the Universidade Federal de Minas Gerais (CEUA/UFMG), under protocol number 83/2021.

## Author contributions

GC, HD, CN, and TV contributed to conception and design of the study. TV, EO, BS, and FS participated in experiments. TV and EO organized the database. EV performed the statistical analysis. TV wrote the first draft of the manuscript. TV and GC wrote sections of the manuscript. All authors contributed to manuscript revision, read, and approved the submitted version.

## Conflict of interest

The authors declare that the research was conducted in the absence of any commercial or financial relationships that could be construed as a potential conflict of interest.

## Publisher's note

All claims expressed in this article are solely those of the authors and do not necessarily represent those of their affiliated organizations, or those of the publisher, the editors and the reviewers. Any product that may be evaluated in this article, or claim that may be made by its manufacturer, is not guaranteed or endorsed by the publisher.
